# Laparoscopic- vs ultrasound-guided TAP block in colorectal surgery. A randomized controlled study

**DOI:** 10.1007/s00464-025-12430-9

**Published:** 2025-12-05

**Authors:** Jonas Nilsson, Felix Bjerregaard, Tobias Pettersson, Ulf O. Gustafsson, Klas Pekkari

**Affiliations:** 1 Department of Clinical Sciences at Danderyd Hospital, Karolinska Institutet, Stockholm, Sweden; 2 Department of Surgery and Urology, Danderyd Hospital, Stockholm, Sweden; 3 Department of Anesthesiology, Danderyd Hospital, Stockholm, Sweden; 4 Department of Molecular Medicine and Surgery, Karolinska Institutet, Stockholm, Sweden; 5 Department of Pelvic Cancer, Division of Coloproctology, Center for Digestive Diseases, Karolinska University Hospital, Solna, Sweden

**Keywords:** TAP block, Colorectal surgery, ERAS, Minimal invasive surgery, Laparoscopic surgery

## Abstract

**Background:**

TAP block is a recommended intervention for postoperative pain relief in colorectal surgery. Although ultrasound TAP (ULTAP) is the most studied approach, a laparoscopic technique (LAPTAP) has been described with heterogeneous results. The objective of this study is to compare laparoscopic-guided transversus abdominis plane (TAP) block versus ultrasound-guided TAP block in minimally invasive colorectal surgery.

**Methods:**

Between May 1, 2021, and July 31, 2023, patients undergoing minimally invasive colorectal surgery were invited to participate in a randomized observer-blind controlled trial at Danderyd Hospital, Sweden. Postoperative pain and analgesia use, along with the time required for the TAP procedure, duration of anesthesia, and surgery were studied. The analysis was conducted on an intention-to-treat basis with subgroup analysis based on participants Body Mass Index (BMI).

**Results:**

A total of 175 participants (88 LAPTAP and 87 ULTAP) underwent randomization. LAPTAP was associated with a shorter anesthesia duration (238 min) than ULTAP (265 min) with a median difference of 27 min (95% CI -49 to -5), and the procedure itself was faster, with a median time of 2 min versus 8 min for ULTAP (median difference -6 min, 95% CI -8 to -4). Pain measured using VAS was similar. There was no significant difference in the mean dosage of postoperative pain medication although more participants in the LAPTAP group than in the ULTAP group received opioids in the postoperative care unit.

**Conclusion:**

LAPTAP is a feasible approach for postoperative pain relief in minimally invasive colorectal surgery, with comparable postoperative pain relief to ULTAP and a significantly shorter time under anesthesia.

The Enhanced Recovery After Surgery (ERAS) protocol in colorectal surgery [1] includes 24 evidence-based interventions aimed at reducing surgical stress and promoting early recovery after surgery. The use of this protocol results in reduced hospital length of stay and morbidity [1–3]. Optimal multimodal analgesia constitutes one of the ERAS interventions, but the evidence in favor of an optimal analgesic regimen is currently weak. The transversus abdominis plane (TAP) block has recently been shown to reduce postoperative pain and opioid consumption, resulting in enhanced mobilization after surgery [4–9].

A TAP block is performed by injecting local anesthetic into the nerve plane between the transverse and oblique muscles of the abdominal wall, thereby blocking the nerves that transmit pain from incisions in this area [10]. The “gold standard” TAP block (ULTAP) is performed by an anesthesiologist using ultrasound to guide the needle into the correct plane of the abdominal wall. However, this procedure can be cumbersome and time-consuming, potentially leading to prolonged anesthesia in patients.

A more recent laparoscopic technique (LAPTAP) without the use of ultrasound has been described to guide the needle to the correct position for the TAP block [11]. This method can be performed by the surgeon during the operation and may shorten anesthesia duration because the method requires less elaborate preparation [12–15].

The use of LAPTAP and ULTAP in minimally invasive colorectal surgery has been previously studied in five randomized controlled studies [13–17]. These studies varied widely in patient populations, timing of TAP block administration during surgery, and dose and concentration of local anesthetics used. Three RCTs showed no difference in postoperative pain and opioid use, whereas LAPTAP was associated with less opioid use in one study. Two previous meta-analyses showed mixed outcomes, highlighting the need for large, well-designed RCTs to further investigate these two methods [18, 19].

The primary outcome in the current study was the difference in postoperative pain measured using a Visual Analog Scale (VAS). Secondary outcomes were consumption of postoperative pain medication, difference in duration of the TAP procedure, duration of surgery and anesthesia, and total time spent in the operating room (OR).

## Materials and methods

### Study design

This was a prospective, single-center, randomized, observer-blind, two-armed study with participants randomized to either standard care ULTAP or intervention LAPTAP at the Danderyd Hospital, Stockholm, Sweden. Between May 1, 2021, and July 31, 2023, all patients who met the inclusion criteria were invited to participate in the study. Adults aged 18 to 85 years scheduled for elective colorectal laparoscopic or robotic-assisted minimally invasive surgery were eligible for enrollment. The exclusion criteria included abdominoperineal resection, documented alcohol or opioid abuse, inability to comply with the Visual Analog Scale (VAS), inflammatory Bowel Disease (IBD), and documented allergy to local anesthetics (ropivacaine). Danderyd Hospital is a designated ERAS Centre of Excellence in Sweden. A standardized ERAS protocol, based on the 2018 ERAS Guidelines [1] was applied to all patients, and all perioperative variables were prospectively registered in the International ERAS Database, EIAS [20]. Patient demographics, clinical data, and study protocol measurements, such as procedure duration and pain evaluation, were consecutively recorded using the REDCap electronic data capture tools hosted at the Karolinska Institute. Pain was assessed using the Visual Analog Scale (VAS), and total pain medication consumption was measured pre- and postoperatively for two consecutive days. Postoperative morbidity was recorded and classified according to the Clavien–Dindo classification system (CD).

Ethical approval was obtained from the Swedish Ethics Committee (DNR: 2020–03845) and the study was registered on clinicaltrials.gov (NCT04907461) prior to patient enrollment.

### Randomization

Participants were randomized (1:1 allocation ratio) into either the intervention or control group in the operating room prior to incision for specimen extraction. Computerized randomization was conducted using the web-based randomization module in REDCap. This randomization was stratified by the type of incision (Phannenstiel or Lateral-Umbilical), and patients were assigned to receive a TAP block either performed by the surgeon using laparoscopic guidance (LAPTAP) or by an anesthesiologist using ultrasound guidance (ULTAP). Patients who underwent a supra umbilical extraction incision or who were converted from laparoscopic to open surgery did not receive a TAP block and were not randomized.

### Intervention

In both groups, the TAP block consisted of 3.75 mg per cc ropivacaine, with a volume of 40 cc divided equally, and injected bilaterally. In the LAPTAP group, the surgeon utilized the laparoscopic technique described by Magee et al. (22). Briefly, the laparoscopic camera was used to visualize the peritoneum and guide the hypodermic needle. Once the needle was visible through the peritoneum, it was retracted by approximately 5 mm. The surgeon then deployed a test dose of the local anesthetics (LA), and after the appearance of the so-called “Doyle’s bulge sign,” the full dose of 20 cc was administered on each side of the abdominal wall in the midaxillary line, between the crista and costal margin. In the ULTAP group, the anesthesiologist performed the TAP block using ultrasound guidance with a high-frequency linear probe following the standard lateral approach. A similar dose of LA was also administered. The same team of surgeons was assigned to both groups and attended a symposium, where they watched an instructional video on the LAPTAP procedure. All anesthesiologists who performed TAP blocks in the study were either specialists or residents in specialist training under the supervision of a specialist. LAPTAP was administered at the end of surgery, after specimen extraction, and just before fascia closure, while ULTAP was performed after surgery, but before the end of anesthesia.

### Evaluation of pre-, intra-, and postoperative pain and analgesia

Postoperative pain was measured according to the VAS scale, where zero indicated an absence of pain and 10 represented the highest imaginable level of pain. VAS recordings were conducted by a ward nurse both at rest and during activity at intervals of two, four, and six hours on the day of surgery (POD0). On postoperative day one (POD1) and two (POD2), the VAS score was measured at rest and during activity at 08:00, 14:00, and 20:00 before the scheduled administration of pain relief medication. The mean of measurements at rest and during activity was calculated for POD0, POD1, and POD2, respectively.

As a standard planned pain relief medication, all participants were offered paracetamol (500 mg), with a maximum of two tablets at four different times per day (02:00, 08:00, 14:00, and 20:00), and Targiniq® (oxycodone hydrochloride + naloxone hydrochloride hydrate), with a maximum of two tablets (5 mg each) twice a day (08:00 and 20:00). Additional pain relief in both the PACU and ward was given upon request for VAS scores of 4 or above. Patients were given either Paracetamol or Oxycodone or both. Analgesics were administered based on individual pain scores and there was no standardized starting dose. For the analysis of postoperative pain medication consumption, the total dosage in mg for each study subject was calculated for the postoperative care unit and ward on POD 0, 1, and 2. For medication on request by participants in the ward, a pooled total of mean consumption in mg for POD 0, 1, and 2 was calculated. To adjust for outliers, the top and bottom 5% were excluded from both VAS and postoperative pain medication.

All VAS scores and pain medications were registered in a dedicated study protocol by a ward nurse who was blinded to whether the study subjects were in the control or intervention group.

### Time measurements in OR

Specific times for the participants’ entry and exit from the operating room, as well as the start and end of anesthesia and surgery, were documented by an OR assistant using a dedicated study protocol. The TAP procedure time was logged when the surgeon or anesthesiologist performing the procedure signaled the start and completion of the TAP block.

### Subgroup analysis

The control and intervention groups were analyzed on an intention-to-treat basis. A per-protocol analysis was conducted to assess whether adherence to the study protocol affected the primary outcome results.

In addition, to analyze the impact of obesity on TAP procedure time and postoperative pain between the groups, the study participants were divided into four subgroups according to the WHO-BMI classification: underweight (BMI < 18.5), normal (BMI = 18.5–24.9), overweight (BMI = 25–29.9), and obese (BMI > 30).

### Data analysis and statistics

A power analysis was conducted on the primary outcome VAS, assuming an estimated detectable difference of one VAS unit (with a standard deviation of 2 in favor of LAPTAP). With 80% power at a two-sided alpha of 0.05, the estimated number of patients needed to be treated was 85 in each group.

For the primary outcome, pain intensity was recorded three times daily using a visual analog scale (VAS, 0–10). For each postoperative day, the mean of the available daily VAS measurements was calculated for each patient to obtain an overall daily pain score. Group differences in median daily pain were analyzed using quantile regression with bootstrap (200 repetitions).

Differences in mean drug consumption were analyzed using linear regression, whereas the odds ratio for receiving opioid treatment in the intervention versus control group was estimated with logistic regression. Median procedure times in the operating room were compared using quantile (median) regression with bootstrap standard errors (200 replications). All data were analyzed using Stata/BE 17.

## Results

**Fig. 1:**
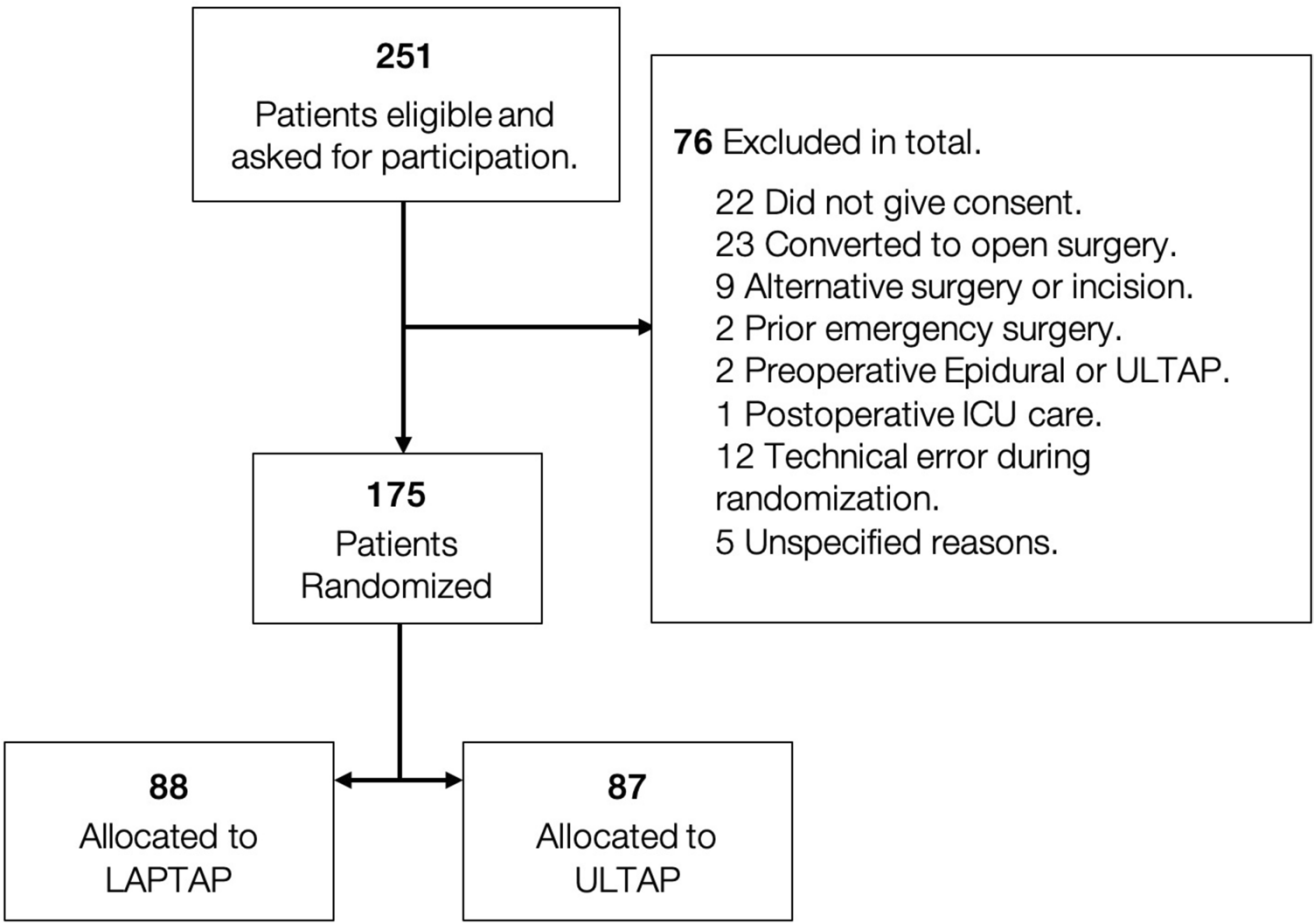
Study flow chart. *LAPTAP* (Intervention) Laparoscopic-guided TAP block. *ULTAP* (Control) Ultrasound-guided TAP block

During the study period, 444 patients underwent minimal invasive colorectal surgery at our hospital. Of these 251 patients met the inclusion criteria, of whom 229 patients provided informed consent. Subsequently, 76 patients were excluded and not randomized for various reasons: 23 underwent conversion to open surgery, 22 did not give consent, nine had either an alteration to the planned surgery or an incision that did not meet the inclusion criteria, two patients underwent emergency surgery before the scheduled procedure, and two were excluded due to preoperative epidural anesthesia or ULTAP procedure. Additionally, one patient experienced intraoperative complications requiring direct transfer to the Intensive Care Unit postoperatively, and 12 were excluded due to technical issues with computerized randomization. Five patients were excluded due to unspecified reasons. A total of 175 patients were randomized, with 88 assigned to the LAPTAP group and 87 to the ULTAP group (Fig. 1).

### Patient characteristics

No statistically significant differences were observed between the control and intervention groups in terms of age, sex, body mass index, ASA-classification, or smoking status. Surgical procedures were comparable between the groups, with a predominance of right hemicolectomy in both groups (56% in LAPTAP and 52% in ULTAP). The number of stomas created (12 in LAPTAP, 13 in ULTAP) and type of incision made for specimen extraction (phannenstiel vs. lateral umbilical, 90% vs. 10% in LAPTAP, and 92% vs. 8% in ULTAP) were similar between the groups (Table 1).

**Table 1:** Baseline characteristics

	LAPTAP, N = 88	ULTAP, N = 87	p-value^*1*^
Age*	73 (66, 78)	73 (64, 80)	0.9
Sex (female, male)	43 (49%), 45 (51%)	49 (56%), 38 (44%)	0.3
BMI*	25 (22, 29)	26 (24, 29)	0.058
ASA			0.8
1	11 (13%)	10 (11%)	
2	51 (58%)	55 (63%)	
3	26 (30%)	22 (25%)	
Smoker n (%)	1 (1.1%)	3 (3.4%)	0.7
Type of surgery			0.5
Rob./Lap. Right hemicolectomy	49 (56%)	45 (52%)	
Rob./Lap. Left hemicolectomy	19 (22%)	21 (24%)	
Rob./Lap. Rectal resection	16 (18%)	21 (24%)	
Rob./Lap. Total colectomy	4 (5%)	0 (0%)	
Stoma created	12 (14%)	13 (15%)	> 0.9
Type of incision			0.6
Phannenstiel	79 (90%)	80 (92%)	
Lateral umbilical	9 (10%)	7 (8.0%)	

^*^Values are median (range)

^1^Wilcox rank sum test

Complications according to the Clavien–Dindo classification for the first 30 postoperative days were similar between the groups for CD grade ≤ 3a, with 15 complications in the control group (17%) and 14 in the intervention group (16%). For complications with a CD grade ≥ 3b, 18 complications (20%) were observed in the intervention group and 4 (5%) in the control group (p = 0.002). This discrepancy was attributed to the higher number of anastomotic leakages in the intervention group (8 vs. 4). No TAP block-related adverse events were recorded in either of the groups.

### Pain according to VAS

Pain according to VAS was similar between the groups on POD 0, POD1, and POD2, respectively. VAS scores at rest varied between 1.5 and 2.3 for LAPTAP and between 1.0 and 2.0 för ULTAP. When participants were in activity, the VAS scores were higher at all time-points for both LAPTAP and ULTAP and varied between 3.3 and 3.0 for LAPTAP and 2.7 and 3.0 for ULTAP. These differences were however not statistically significant (Table 2).

**Table 2:** VAS score, intention to treat

		ULTAP, N = 87	LAPTAP, N = 88	
		**Median** (IQR)^1^	**Median** (IQR)^1^	**Median difference** (95% CI)^2^
POD 0	VAS at rest	2.0 (0.7 – 3)	2.3 (1 – 3.5)	0.3 (−0.2 – 0.9)
	VAS activity	2.7 (1.3 – 4)	3.3 (1.5 – 4.5)	0.7 (−0.3 – 1.4)
POD 1	VAS at rest	1.7 (0.3 – 3)	1.5 (0.6 – 2.7)	0.2 (−1.1 – 0.7)
	VAS activity	3.0 (1.7 – 4)	3.0 (1.7 – 4.3)	0.0 (−1.0 – 1.0)
POD 2	VAS at rest	1.0 (0 – 2.3)	1.5 (0.3 – 2.5)	0.5 (−0.3 – 1.3)
	VAS activity	2.7 (1.7 – 4)	3.0 (2 – 4)	0.3 (−0.3 – 0.9)

^1^Median (Interquartile Range), adjusted for 5% outliers on both ends

^2^Effect size expressed as mean difference (LAPTAP—ULTAP) with 95% Confidence Interval

### Pain medication

All participants were administered a standard preoperative pain medication regimen of 1500 mg paracetamol, 10 mg oxycodone, and 200 mg celecoxib. Two patients in each group were administered a single dose of clonidine. Analgesic administration during surgery did not differ between groups (data not shown).

No significant difference was observed in the mean dosage of pain medications in the postoperative care unit. However, in the LAPTAP group, a significantly higher number of participants received pain relief in the form of morphine: 52 (59%) for LAPTAP and 36 (41%) for ULTAP (OR 2.1, 95% CI 1.1 to 3.7). When all opioids (Morphine, Oxycodone, Ketobemidone, and Fentanyl) were pooled together, the number of patients in the LAPTAP who received any opioid was 74 (65%) vs. 51 (44%) for the ULTAP (OR 2.8, 95% CI 1.5 to 5.2).

Planned paracetamol and oxycodone consumption were similar on postoperative days 0, 1, and 2 for both ULTAP and LAPTAP. The additional analgesics requested by the participants showed no significant differences among the groups (Table 3).

**Table 3:** Postoperative pain medication, intention to treat

	ULTAP, N = 87		LAPTAP, N = 88		
	**Mean** (SD)^1^	**n**^2^	**Mean** (SD)^1^	**n**^2^	**Mean difference** (95% CI)^3^
**Postoperative Care Unit**					
Morphine (mg)	2.1 (3.1)	87	2.8 (3.1)	84	0.68 (−0.3 – 1.6)
Paracetamol (mg)	500.0 (502.9)	86	522.7 (502.3)	88	22.7 (−127.7 – 607)
Clonidine (µg)	20.1 (32.9)	86	27.0 (35.2)	85	6.9 (−3.3 – 17.2)
Ketobemidon (mg)	0.5 (1.5)	86	0.9 (1.9)	87	0.4 (−0.1 – 0.9)
Oxycodone (mg)	0.5 (1.5)	86	0.8 1.8)	85	0.3 (−0.2 – 0–8)
Parecoxib (mg)	2.3 (9.4)	87	5.2 (13.4)	88	2.9 (−0.5 – 6.4)
**Postoperative Ward, Planned Medication**					
**POD 0**					
Paracetamol (mg)	811.8 (393.2)	85	761.9 (428.5)	84	−49.9 (−174.7 – 75.0)
Oxycodone (mg)	4.6 (1.3)	84	4.3 (1.8)	88	−0.38 (−0.8 – 0.1)
**POD 1**					
Paracetamol (mg)	3058.8 (446.0)	85	3006.0 (399.5)	84	−52.9 (−181.5 – 75.8)
Oxycodone (mg)	9.8 (1.0)	77	9.5 (1.5)	84	−0.3 (−0.7 – 0.1)
**POD 2**					
Paracetamol (mg)	2689.7 (815.0)	87	2517.1 (1026.8)	88	−172.6 (−449.4 – 104.2)
Oxycodone (mg)	8.4 (3.2)	83	7.8 (3.5)	84	−0.6 (−1.6 – 0.5)
**Postoperative Ward, On Demand**					
**POD 0**					
Paracetamol (mg)	69.0 (254.9)	86	45.5 (209.5)	87	−23.5 (−93.1 – 46.1)
Oxycodone (mg)	1.4 (2.5)	87	1.5 (2.9)	88	0.1 (−0.7 – 0.9)
**POD 1**					
Paracetamol (mg)	34.5 (183.5)	87	80.5 (273.6)	87	46.0 (−23.7 – 115.7)
Oxycodone (mg)	4.1 (5.5)	84	5.5 (6.7)	87	1.4 (−0.4 – 3.3)
**POD 2**					
Paracetamol (mg)	103.5 (306.3)	87	160.9 (369.6)	87	57.5 (−44.1 – 159.0)
Oxycodone (mg)	3.1 (5.6)	86	3.1 (4.9)	86	0.1 (−1.5 – 1.7)

^1^Mean (Standard Deviation)

^2^Number of observations when adjusted for 5% outliers on both ends

^3^Effect size expressed as mean difference (LAPTAP—ULTAP) with 95% Confidence Interval

### Time differences of procedures in the OR

Participants in the LAPTAP group experienced a shorter duration of general anesthesia than those in the ULTAP group. The median anesthesia time for LAPTAP was 238 min compared to 265 min for ULTAP corresponding to a median difference of −27 min (95% CI −49 to −5). The LAPTAP procedure was less time-consuming, with a median time of 2 min compared to 8 min for ULTAP (median difference −6 min, 95% CI −8 to −4). The total time the participants spent in the operating theater was shorter for LAPTAP with a median of 266 min compared to ULTAP 286 min (median difference −20 min, 95% CI −43 to 3), although the difference was not statistically significant. The addition of the LAPTAP procedure during the surgical procedure, in contrast to ULTAP performed after surgery but under anesthesia, did not appear to affect the duration of surgery with a median duration of 176 min for LAPTAP and 191 min for ULTAP (median difference −15 min, 95% CI −32 to 3) (Table 4).

**Table 4:** Time measures, intention to treat

	ULTAP, N = 87	LAPTAP, N = 88	
	**Median** (IQR)^1^	**Median** (IQR)^1^	**Median Difference** (95% CI)^2^
**Time in OR** (min)	286 (244 – 330)	266 (230 – 318)	− 20 (−43 – 3)
**Anesthesia** (min)	265 (223 – 302)	238 (198 – 285.5)	− 27 (−49 – −5)
**Surgical procedure** (min)	191 (151 – 229)	176 (150.5 – 233)	− 15 (−33 – 3)
**TAP procedure** (min)	8.0 (5 – 10))	2.0 (1 – 3)	− 6 (−8 – −4)

^1^Median (Interquartile Range)

^2^Effect size expressed as median difference (LAPTAP—ULTAP) with 95% Confidence Interval

### Per-protocol analysis

A per-protocol analysis was performed after excluding nine participants from the LAPTAP group and six from the ULTAP group, who were unable to follow the study protocol postoperatively. Eight patients underwent early reoperation or ICU care. Four participants were unable to perform ULTAP; One due to obesity, two due to subcutaneous gas, and one due to right-sided TAP (reason not specified). Two participants were excluded due to early discharge (POD1), and one patient was excluded due to prescription of opioids 10 days before surgery.

Among these patients, there was a difference in postoperative VAS score in motion at POD 0 with 3.3 for LAPTAP compared to 2.3 for ULTAP (median diff. 1.0, 95% CI 0.2 to 1.8). No significant differences were found in amount of pain medication administered between the LAPTAP and ULTAP groups on POD 0, 1, and 2.

### Effect of obesity

Only three study participants, all in the LAPTAP group, had an BMI under 18.5 why no analysis could be conducted for this subgroup. The TAP procedure time correlated with the results for the intention-to-treat cohort, with a significantly shorter procedure time in the LAPTAP group for each BMI subgroup.

There were no significant differences in VAS scores at rest between participants on any day across any BMI group when comparing the LAPTAP and ULTAP groups. However, for participants with a BMI of 25–29.9, differences in the VAS scores during motion were observed. On POD 1, the median VAS scores in motion were 2.3 for ULTAP and 3.7 for LAPTAP (median diff. 1.33, 95% CI 0.3 to 2.4). No significant differences were found in motion on POD 0 or 2 in any of the BMI groups.

## Discussion

This single-center randomized controlled observer-blind study compared laparoscopic (LAPTAP) and ultrasound-guided (ULTAP) transversus abdominis plane blockade in minimally invasive colorectal surgery. No difference was observed in postoperative pain on the day of surgery or within 48 h after surgery. The mean consumption of postoperative pain medication was similar between the two groups; however, a higher number of patients in the LAPTAP group received opioids during the immediate postoperative period in the postoperative care unit compared to the ULTAP group. On postoperative days 1 and 2 in the ward, the mean consumption of analgesics and the number of patients requesting on-demand opioids did not differ between the groups. In addition, we found that laparoscopic-guided TAP was associated with a shorter time of anesthesia and shorter procedure time, but equal time of surgery.

The TAP blockade has been well studied as a method for postoperative pain relief in colorectal surgery. Liu et al. published a meta-analysis of 14 RCTs comparing TAP blockade versus no TAP blockade, which showed less postoperative pain at rest and in motion, and less postoperative opioids in favor of TAP blockade [21]. Ultrasound-guided TAP was used for TAP blockade in 13 of the 14 studies included in the meta-analysis. When TAP blockade was compared with epidural analgesia in an ERAS setting, no difference was observed for postoperative analgesia, but TAP had fewer urinary retention complications than epidural analgesia [22].

LAPTAP and ULTAP have been compared in five RCTs, each comprising 60–340 patients [13–17]. One study, Zaghiyan et al., showed less postoperative opioid use after LAPTAP than after ULTAP. One caveat of this three-armed study is that a difference in opioid use was detected only between LAPTAP and ULTAP, with no difference observed between the ULTAP and the group that did not receive TAP blockade. Additionally, the postoperative observer was not blinded to the intervention that the patient had received. A further limitation is that the study population differed from ours, as more than 50% of participants underwent surgery for inflammatory bowel disease, and almost 20% had a proctectomy. As IBD patients are well known for complex pain issues, both IBD and proctectomy were excluded from our study [13]. The largest RCT to date published by Salomonsen et al. comparing LAPTAP and ULTAP in colorectal patients included 340 patients in three arms [17]. Our study show the same results concerning differences in postoperative opioid consumption and procedure time for LAPTAP vs ULTAP. In contrast to the study by Salomonsen et al. we did not include patients with upper midline incisions and patients who were converted to open surgery. In addition, Salomonsen et al. used a dual subcostal TAP block technique not used in any of the other published RCTs thereby limiting their comparability with other studies.

Our results are in line with four other RCTs where three showed no difference in postoperative pain and opioid use, and one showed no difference in pain but a larger number of patients with on-request opioids [14–16].

Perceived pain according to the VAS was measured before administering opioids or other pain medications to the patients. We found no significant difference in the VAS scores or mean postoperative opioid doses between the two groups. However, a larger proportion of the patients in the LAPTAP group (65% vs. 44%) received opioids in the postoperative care unit. Several factors may explain the current results and variability across studies. One possible factor is the timing of the TAP block, as some studies administer TAP at the beginning of surgery, while others do so at the end. In our study, LAPTAP was administered at the end of surgery, after specimen extraction, and just before fascia closure, while ULTAP was performed after surgery, but before the end of anesthesia. Additionally, studies vary in the volume, type, and concentration of the local anesthetic used for TAP block. In our study, 20 ml of 0.375% ropivacaine was injected bilaterally, whereas other studies have used bupivacaine or ropivacaine in volumes up to 30 ml bilaterally and concentrations as low as 0.2%. The difference in timing between LAPTAP and ULTAP can also be a potential source of bias. However, given the pharmacokinetics of local anesthetics and the typical duration of action of TAP blocks (several hours), this short interval (median 28 min) is unlikely to have had any clinically relevant impact on analgesic efficacy or pain scores. Furthermore, all other perioperative variables were standardized according to ERAS guidelines, minimizing potential bias related to timing.

We showed that LAPTAP was associated with 27 min less anesthesia than ULTAP. This is important because prolonged anesthesia is associated with increased morbidity [23].

The single-center study design in a Scandinavian setting limits the generalizability of this finding. In Scandinavia an anesthesiologist is typically present only during the initial phase for sedation and intubation. During the remainder of the surgery, a specialized nurse is responsible for maintaining anesthesia. This affects the workflow for ULTAP, as the anesthesiologist must be notified and arrive in time for the procedure, making the process more logistically challenging and potentially increasing both operative and procedure times. This might not be the case when the anesthesiologist is present during the whole procedure.

No TAP procedure-related complications were observed in either the LAPTAP or ULTAP group. However, severe postoperative complications were more frequent in the LAPTAP group, primarily due to a higher incidence of anastomotic leakage (9% vs. 5% in the ULTAP group). This difference could not be attributed to variations in the surgical procedures. Both groups had similar rates of rectal resection (18% LAPTAP vs. 24% ULTAP), nor was it related to smoking status or ASA classification. The TAP block technique itself is highly unlikely to have contributed causally to these events. TAP blocks are performed in the abdominal wall and are not associated with physiological effects that could influence anastomotic healing, hemodynamics, or systemic inflammatory responses. Importantly, no TAP block–related adverse events were recorded in either group.

Our study was not powered for a subgroup analysis. However, we performed an analysis in which we divided the participants into three groups depending on their BMI. This was done because we speculated that this could be an advantage of LAPTAP in obese participants. We did not find any differences in the VAS scores at rest. For the two groups with BMI < 30, ULTAP seemed to have a better effect when participants were in motion on postoperative day 1. No difference was observed in patients with BMI > 30 or on postoperative day 2. The interpretation of this analysis should be made with caution, since we only had 23–33 participants in each group. Especially for ULTAP, inter-operator variability is an important consideration and particularly in patients with higher BMI where visualization and access can be more challenging. However, formal validation of technical accuracy or assessment of inter-operator variability was not feasible within the scope of this study. The ULTAP blocks were however performed by experienced anesthesiologists who were either specialists or residents under direct supervision of a specialist, and the procedures were standardized according to departmental guidelines. This minimizes, though does not completely eliminate, potential operator-dependent variability.

A limitation of our study is its single-center design, and we did not assess the effect of TAP block in the postoperative care unit using a standardized test. For statistical analysis, we used quantile regression to compare postoperative pain scores between the intervention and control groups at each postoperative day. Quantile regression is well suited for non-normally distributed outcomes such as VAS pain scores and provide a robust estimate of group differences. However, because pain was measured repeatedly over time and our approach did not account for within-patient correlation, one could argue that a longitudinal approach, such as a linear mixed-effects model, would provide a more robust framework. However, the study’s strengths include being one of the largest RCTs conducted to date with a well-defined study population, observer-blind design, and implementation within an ERAS protocol. Ensuring that all patients followed the protocol helped establish a standardized level of care, creating an environment particularly well suited for conducting an RCT. Additionally, we stratified the control and intervention groups based on the type of the specimen extraction site.

We conclude that LAPTAP is a safe method for postoperative analgesia in minimally invasive colorectal surgeries. The effect is comparable to that of ULTAP, with the benefits of being less time consuming.

## Data Availability

Due to Swedish legal restrictions and the current ethical approval for the study, data are not publicly available to share, but the research group can provide descriptive data in a table form.
